# A single-cell mass cytometry platform to map the effects of preclinical drugs on cartilage homeostasis

**DOI:** 10.1172/jci.insight.160702

**Published:** 2022-10-24

**Authors:** Neety Sahu, Fiorella Carla Grandi, Nidhi Bhutani

**Affiliations:** Department of Orthopedic Surgery, School of Medicine, Stanford University, Stanford, California, USA.

**Keywords:** Cell Biology, Therapeutics, Osteoarthritis

## Abstract

No disease-modifying drug exists for osteoarthritis (OA). Despite success in animal models, candidate drugs continue to fail in clinical trials owing to the unmapped interpatient heterogeneity and disease complexity. We used a single-cell platform based on cytometry by time-of-flight (cyTOF) to precisely outline the effects of candidate drugs on human OA chondrocytes. OA chondrocytes harvested from patients undergoing total knee arthroplasty were treated with 2 drugs, an NF-κB pathway inhibitor, BMS-345541, and a chondroinductive small molecule, kartogenin, that showed preclinical success in animal models for OA. cyTOF conducted with 30 metal isotope–labeled antibodies parsed the effects of the drugs on inflammatory, senescent, and chondroprogenitor cell populations. The NF-κB pathway inhibition decreased the expression of p–NF-κB, HIF2A, and inducible NOS in multiple chondrocyte clusters and significantly depleted 4 p16^ink4a^-expressing senescent populations, including NOTCH1^+^STRO1^+^ chondroprogenitor cells. While kartogenin also affected select p16^ink4a^-expressing senescent clusters, there was a less discernible effect on chondroprogenitor cell populations. Overall, BMS-345541 elicited a uniform drug response in all patients, while only a few responded to kartogenin. These studies demonstrate that a single-cell cyTOF-based drug screening platform can provide insights into patient response assessment and patient stratification.

## Graphical abstract

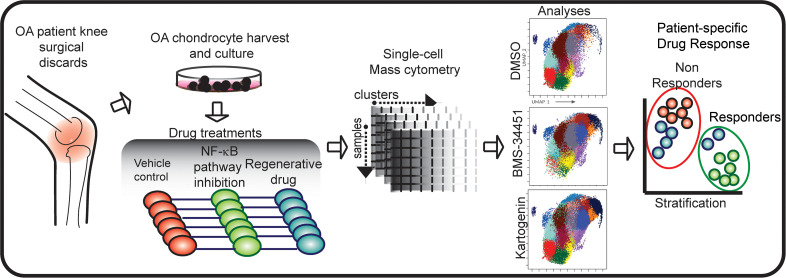

## Introduction

Cartilage repair remains an unmet medical need with no disease-modifying drug available for degenerative diseases like osteoarthritis (OA), for which clinical care is limited to pain management or total joint replacement. Although multiple pathways and targets have been considered for OA treatment, the rate of drug failure in clinical trials is high. Notably, OA is a complex disease with a known interplay of metabolic (1), epigenetic (2, 3), genetic (4), and cellular factors (5, 6) that influence its etiology. Drug discovery efforts are further confounded by the late detection of the disease and ambiguity about the causal and early events in OA pathogenesis. Another critical knowledge gap is the lack of understanding of the heterogeneity between patients and its molecular underpinnings.

The recent advent and accessibility of single-cell technologies, both transcriptional and proteomic, have made it possible to achieve a high-resolution understanding of the composition, architecture, and functioning of tissues by parsing out the contribution of single cells in tissues in health and disease (7, 8). Using a single-cell mass cytometry approach, we recently built a cellular atlas for human cartilage in healthy and OA patients wherein we defined a set of regenerative chondroprogenitor cell populations as well as new cartilage-intrinsic immunomodulatory populations (9). Our cytometry by time-of-flight (cyTOF) analyses identified and delineated 3 distinct chondroprogenitor cell (CPC) subtypes: CPC I (CD105^lo^), which were enriched in normal chondrocytes; CPC II, which were present in both normal and OA chondrocytes; and CPC III, which were enriched in OA chondrocytes (inflamed CD105^hi^). Additionally, we identified an inflammation-amplifying population termed Inf-A, defined by elevated levels of IL1R1 (CD121A) and TNFRII (CD120B) receptors. TNFRII has been shown to have antiinflammatory effects as opposed to TNFRI in some cell types (10). Chondrocytes coexpressing IL1R1 (CD121A) and TNFRII (CD120B), however, showed active signaling through SMAD1/5 and JNK pathways, and inhibition of either of these pathways dampened secretion of inflammatory cytokines (9). Another functional, inflammation-dampening population, Inf-D, was characterized by the expression of CD24, a molecule we had previously identified as a negative regulator of NF-κB–mediated inflammation. Activation of Inf-D in combination with Inf-A inhibition affected multiple cytokines for an immunomodulatory effect on OA cartilage secretome.

Given this new understanding of the cartilage landscape, we have now utilized the power of cyTOF to test how a given drug affects these precise chondrocyte subpopulations and their crosstalk to get a high-resolution snapshot rather than just the cumulative effect on the total population. A huge knowledge gap exists between studies in rodent or large-animal models and the ultimate clinical trials in humans. We postulate that a single-cell-based screening platform for patient chondrocyte samples, with its high resolution, may be able to provide more insightful analyses for effective screening approaches for OA therapeutics before clinical trials.

## Results

**Figure 1:**
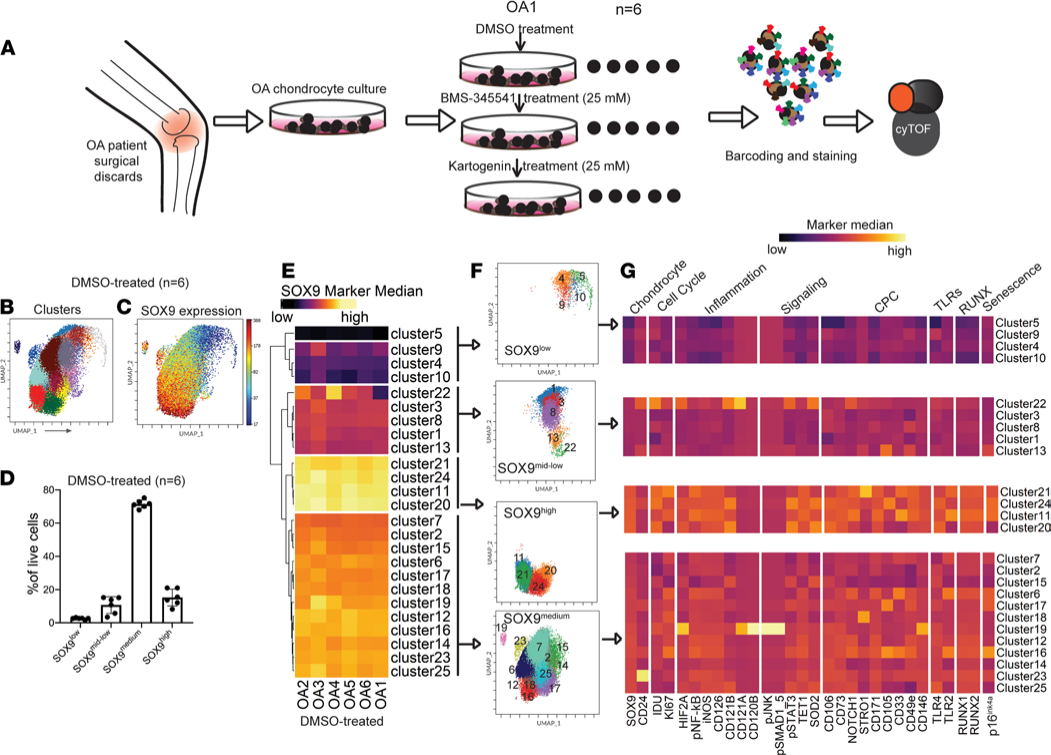
Stratification of OA chondrocytes based on SOX9 expression. (**A**) Schematic outlining the experimental design for profiling OA chondrocytes. Briefly, OA chondrocytes were isolated from patient samples (*n* = 6), cultured, and treated with DMSO, BMS-345541 (25 μM), or kartogenin (25 μM) for 48 hours followed by staining with metal-conjugated antibodies and data acquisition using cyTOF. (**B** and **C**) UMAP of 25 clusters identified by unsupervised hierarchical clustering by FlowSOM is shown for concatenated DMSO-treated samples (*n* = 8,043 cells per sample) (**B**) juxtaposed with UMAP of the median SOX9 expression across all clusters (**C**). (**D**) Bar plot shows the abundance of the 4 groups stratified by median SOX9 expression in DMSO-treated controls. Each point represents an individual patient (*n* = 6). Data represent mean ± SD. (**E**) Heatmap with hierarchical clustering with Euclidean distance measurement for stratification of clusters based on SOX9 expression. (**F**) UMAP representation of the SOX9^lo^, SOX9^lo/mid^, SOX9^mid^, and SOX9^hi^ groups. (**G**) Heatmap for the median expression of various panel markers in the groups. Marker columns were *z*-scored for standardization.

We have used cyTOF, a mass spectrometry–based high-dimensional method for single-cell detection of isotope-labeled antibodies, to stain OA chondrocytes (Figure 1A). In our previous study (9), we differentiated OA chondrocytes from 20 different patients into 3 subtypes, groups A, B, and C, based on the differential abundance of clusters of CPCs. Group A, enriched for CD105^+^ CPCs, was the largest (12 of 20 patients) compared with groups B and C in the OA cohort. In the current study, we chose 6 OA patient samples from group A where OA chondrocytes were available in sufficient numbers for us to perform high-resolution mapping by cyTOF to study the effect of preclinical drugs. Chondrocytes were treated with control (DMSO), an NF-κB pathway inhibitor (BMS-345541), or a regenerative drug candidate (kartogenin) for 48 hours, fixed, barcoded, stained, and profiled together by cyTOF (Figure 1A).

Previously, we had optimized a panel of 33 markers for profiling chondrocytes, including cell surface receptors, adhesion molecules, signaling mediators, and cell cycle and transcription factors. For the present study, we retained most of the previous markers, such as the cytokine receptors IL1R1 (CD121A) and TNFRII (CD120B), which defined an inflammation-amplifying population (Inf-A); CD24, which characterized an inflammation-dampening (Inf-D) subpopulation; and multiple CPC markers, including CD105, CD73, CD106, NOTCH1, and STRO1 (30 markers total) (Supplemental Table 1; supplemental material available online with this article; https://doi.org/10.1172/jci.insight.160702DS1). We have refined the panel by excluding redundant markers and including markers like p16^ink4a^ that have been reported to identify a senescent population in OA cartilage (11) and markers like the Toll-like receptors TLR2 and TLR4 that play roles in innate immunity (12). Additionally, in our previous study, we discarded cells with low expression of SOX9 (~10%–13% of the total chondrocyte population). With the rationale that these cells can constitute putative stem/progenitor cells or rare infiltrating stromal or immune cells and should be characterized, we have now included these SOX9^lo/mid^ cells in our analyses using SOX9 as a stratification marker for their identification.

Each set of chondrocytes (*n* = 6) per treatment was barcoded to allow multiplexing and reduce staining variability between samples. The barcoded samples were stained together with metal-conjugated antibodies, and then cyTOF was performed. Following data acquisition, the data samples were debarcoded using the premessa package in R (https://github.com/ParkerICI/premessa). The individual data sets from each sample corresponding with treatments (DMSO, BMS-345541, and kartogenin) were uploaded into the Cytobank platform (Beckman Coulter Life Sciences) for data analysis.

For clustering using self-organizing map by FlowSOM (https://support.cytobank.org/hc/en-us/articles/360018965212-Introduction-to-FlowSOM-in-Cytobank), we used the parameters that were optimized in our previous study, i.e., a cluster number of 25 and a metacluster number of 10 in the Cytobank platform (9). The optimal cluster and metacluster numbers (25 and 10, respectively) were selected after calibration and deemed sufficient in resolving the clusters (each cluster should have non-zero and greater than 1,000 events across all samples) and identifying distinct subpopulations. Uniform manifold approximation and projection (UMAP) for individual control samples (*n* = 6, DMSO-treated) was concatenated and is presented in Figure 1B. UMAP of clusters in each sample as well as the cluster proportions in all samples (DMSO-treated, BMS-345541–treated, and kartogenin-treated) is presented in Supplemental Figure 1.

### Characteristics of SOX9^lo/mid^ cell populations in OA cartilage.

Based on the median expression levels of SOX9 in the 6 DMSO-treated samples, all clusters were stratified into 4 groups using hierarchical clustering: SOX9^lo^, SOX9^lo/mid^, SOX9^mid^, and SOX9^hi^ (Figure 1, C–F). The SOX9^lo^ group consisted of 4 clusters (4, 5, 9, and 10) and comprised only 2.6% of the total live cells (Figure 1D). The SOX9^lo/mid^ group consisted of 5 clusters (1, 3, 8, 13, and 22) and comprised 10.7% of the total live cells. Together, these cells that were excluded from analyses in our previous study comprised 13.3% of cells, hence were overall a small fraction of the OA chondrocytes. Interestingly, the clusters in the SOX9^lo^ group were distinct from all other groups in having quite low median expression levels of the markers of CPCs or inflammatory populations, suggesting that these were either OA chondrocytes with low inflammation or chondrocytes undergoing dedifferentiation (Figure 1G). In contrast, the SOX9^lo/mid^ group was like the previously profiled SOX9^mid^ and SOX9^hi^ groups in consisting of clusters that either had CPC-like phenotype (like CD105^+^ cluster 13) or had high inflammation (like cluster 22 with the expression of the IL-1β receptors CD121A and CD121B). The SOX9^mid^ group was the largest, comprising 71.5% of total live cells, while the SOX9^hi^ group comprised the remaining 15%. Interestingly, SOX9^hi^ clusters (11, 20, 21, and 24) appeared highly inflamed with a higher overall expression of HIF2A, p–NF-κB, inducible NOS (iNOS), the IL-6 receptor CD126, and the IL-1β receptor IL1R1 (CD121A). The SOX9^mid^ group, on the other hand, was mixed, consisting of clusters with lower levels of inflammation (clusters 2, 7, and 15) along with clusters showing higher levels of p–NF-κB, iNOS, and the IL-6 receptor CD126 (clusters 6, 12, and 16).

### p16^ink4a^-expressing senescent cell populations in OA chondrocytes are distinct from Inf-A cells.

**Figure 2:**
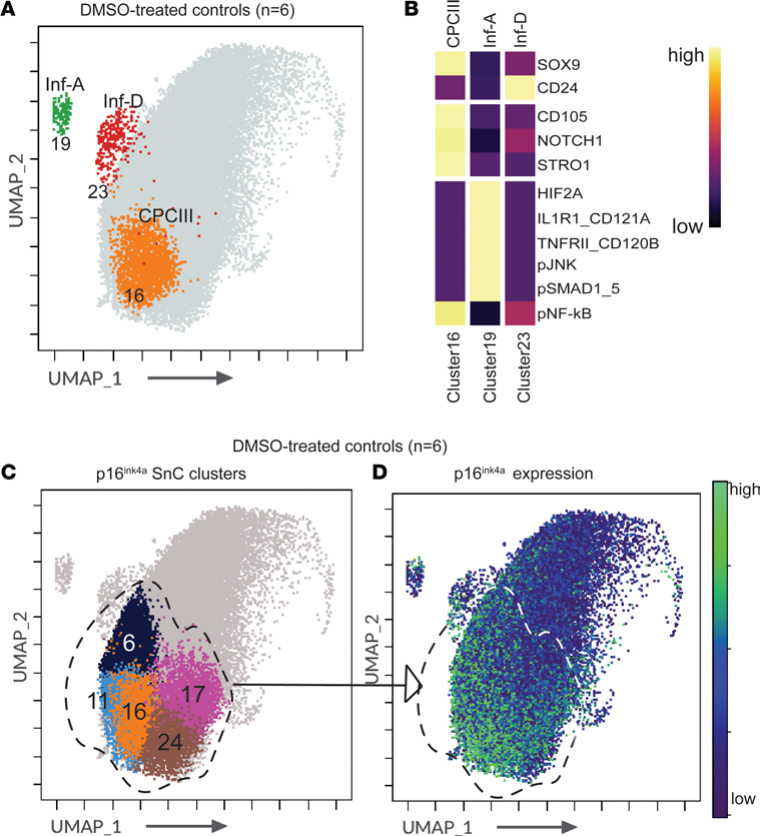
Identification of inflammatory and senescent populations in the OA chondrocyte landscape. (**A**) UMAP demarcating Inf-A (cluster 19), Inf-D (cluster 23), and CPC III (cluster 16) populations in OA chondrocytes (*n* = 6 biological samples, 8,043 cells per sample concatenated). (**B**) Heatmap shows median expression of markers used to precisely identify CPC III (CD105^+^NOTCH1^+^STRO1^+^), Inf-A (HIF2A^+^CD121A^+^CD120B^+^p–NF-κB^+^p-JNK^+^p-SMAD1/5^+^), and Inf-D (CD24^+^) clusters. (**C**) UMAP projection showing p16^ink4a^-expressing senescent clusters (SnC). (**D**) UMAP of the median expression of p16^ink4a^ in OA chondrocytes (*n* = 6). The area demarcated by a dashed line denotes the juxtaposition of the p16^ink4a^ clusters on the p16^ink4a^ expression map.

Our previously reported subpopulations in OA cartilage — the CD105^+^NOTCH1^+^STRO1^+^ cartilage-progenitor population termed CPC III; the inflammation-amplifying population termed Inf-A, defined by coexpression of IL1R1 (CD121A) and TNFRII (CD120B) receptors; and the CD24^+^ Inf-D — were all identified in this study. Clusters 19, 23, and 16 were identified as the Inf-A, Inf-D, and CPC III populations, respectively, all belonging to the SOX9^mid^ group (Figure 2, A and B, and Supplemental Table 2).

p16^ink4a^ was previously identified to mark a functional senescent population in OA cartilage that, upon ablation or inhibition, showed a decrease in senescence-associated secretory phenotype (SASP) and delayed OA in a mouse model (11). We therefore investigated p16^ink4a^ expression, and found it to be quite widespread in OA chondrocytes and not restricted to a very small population. FlowSOM identified 5 different clusters that expressed high levels of p16^ink4a^ — clusters 6, 11, 16, 17, and 24 (Figure 2, C and D). Of these, clusters 6, 16, and 17 belonged to the SOX9^mid^ group, while clusters 11 and 24 belonged to the SOX9^hi^ group. Interestingly, no p16^ink4a^-expressing senescent subpopulations were detected in the SOX9^lo^ or the SOX9^lo/mid^ group. It is also interesting that the Inf-A population was distinct from the senescent populations identified by FlowSOM, although a few Inf-A cells did express p16^ink4a^. This observation suggests that Inf-A cells provide distinct contributions to the inflammatory microenvironment of cartilage in addition to the contribution to the SASP by p16^ink4a^-expressing cells. We also noted that the CPC III population (cluster 16) showed high p16^ink4a^ and was one of the senescent clusters (hereafter referred to as SnC CPC III). An independent study postulated that CPCs can undergo senescence in OA cartilage (13). SnC CPC III cells also expressed CD33 and TLR2 (Supplemental Figure 2), markers that are also coexpressed with p16^ink4a^ in clusters 6 and 11 (labeled SnC I and SnC II). The other 2 clusters, 17 and 24 (labeled SnC III and SnC IV), showed higher expression of CD105 and p16^ink4a^ but not of CD33, TLR2, NOTCH1, or STRO1 (Supplemental Figure 2).

### NF-κB inhibition greatly alters the OA landscape by depleting p16^ink4a^-expressing senescent clusters.

The NF-κB pathway is known to play a significant role in the function of inflammatory cytokines including IL-1β and TNF-α in OA pathogenesis, and drugs targeting this pathway have been extensively investigated (14–16). Since activation of inhibitor of NF-κB (IκB) kinase is essential for the phosphorylation of NF-κB for its activation, we used a known selective inhibitor of IκB kinase, BMS-345541, to test the precise effect of NF-κB pathway inhibition. The inhibitor BMS-345541 has already been shown to be effective in inhibiting OA pathogenesis in a preclinical mouse model of OA (17).

**Figure 3:**
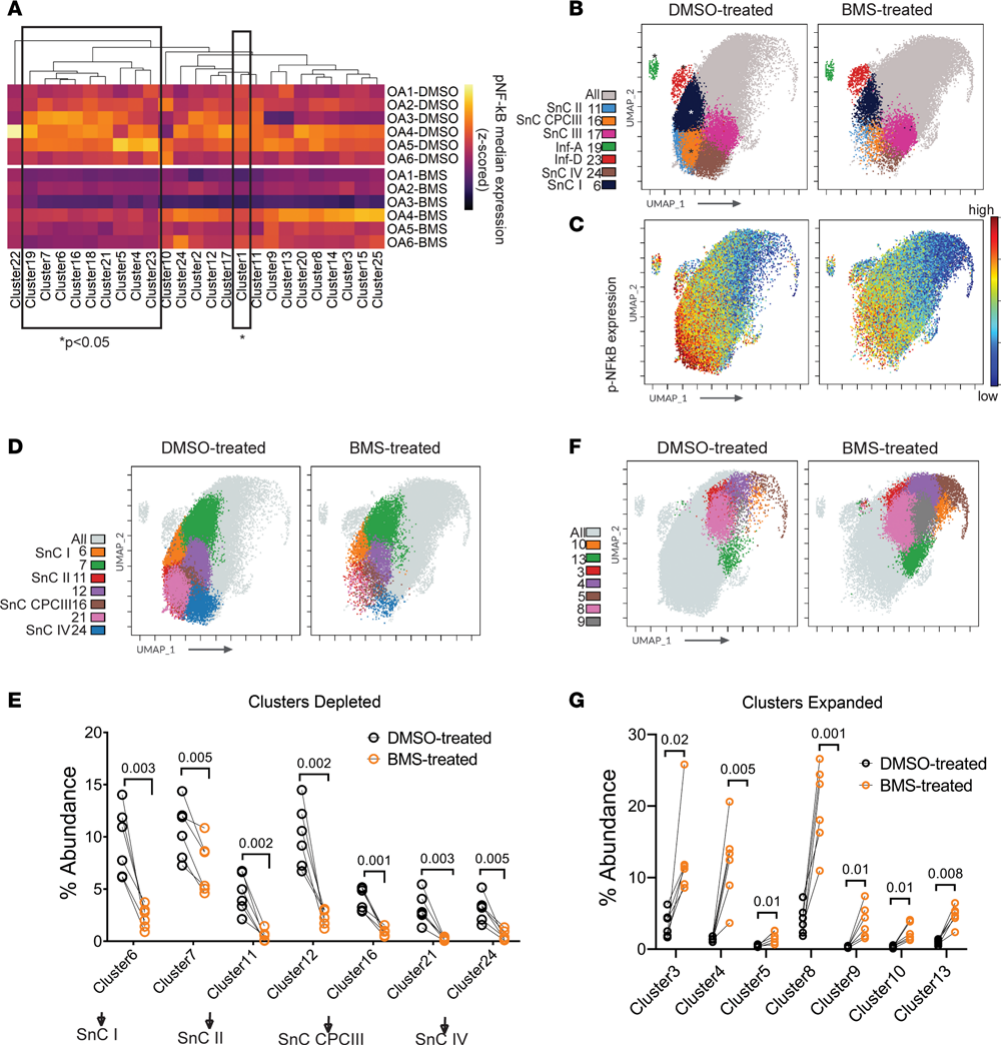
Effect of NF-κB pathway inhibition on the OA chondrocyte landscape. (**A**) Heatmap of median p–NF-κB expression in all FlowSOM-identified clusters in DMSO-treated (control) and BMS-345541–treated OA chondrocytes (*n* = 6 patient samples per treatment). p–NF-κB expression in each cluster was column-wise *z*-scored for standardization. Rectangles denote significantly different clusters between control and BMS-345541–treated samples measured by paired *t* test (*P* < 0.05). (**B**) UMAP projections of Inf-A (cluster 19), Inf-D (cluster 23), and CPC III (cluster 16) populations, along with p16^ink4a^-expressing senescent clusters: SnC I (cluster 6), SnC II (cluster 11), SnC III (cluster 17), and SnC IV (cluster 24) in control and BMS-345541–treated samples (*n* = 6 samples concatenated). Asterisks indicate clusters with significantly decreased expression of p–NF-κB upon BMS-345541 treatment. (**C**) UMAP represents median p–NF-κB expression across OA landscape in control and BMS-345541–treated samples (*n* = 6). (**D** and **F**) UMAP of significantly depleted or expanded clusters upon BMS-345541 treatment. (**E** and **G**) Percentage abundance of significantly depleted or expanded clusters in paired samples (*n* = 6). Statistical significance was calculated by paired *t* test (*n* = 6) at 95% confidence level and represented by adjusted *P* values.

On average, there was variable inhibition of NF-κB pathway (phospho–serine 29) in different clusters of individual OA patients, with only 10 of the clusters demonstrating a significant downregulation (Figure 3A). Notably, p–NF-κB expression was significantly reduced in Inf-A (cluster 19), Inf-D (cluster 23), and SnC CPC III (cluster 16) clusters along with SnC I (cluster 6) (Figure 3, B and C). The expression of inflammatory iNOS and HIF2A, directly regulated by p–NF-κB, was also significantly reduced in select clusters, with all 3 markers being downregulated only in Inf-A and Inf-D clusters (Supplemental Figure 3).

**Figure 4:**
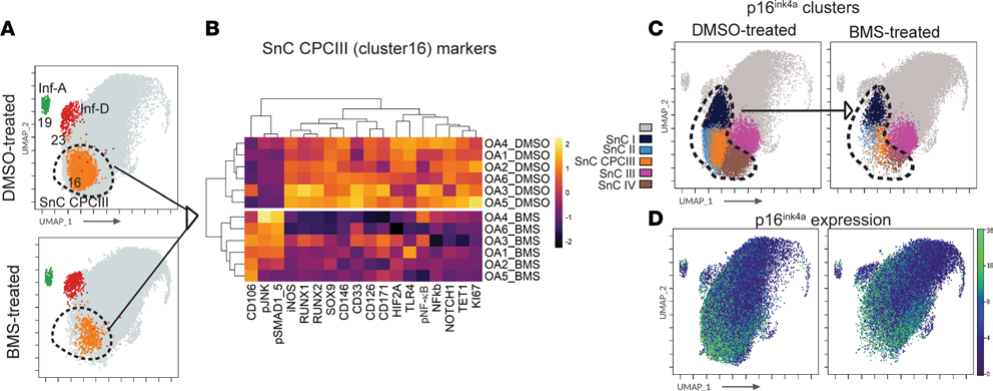
NF-κB pathway inhibition targets p16^ink4a^-expressing senescent cells. (**A**) UMAP of Inf-A, Inf-D, and Snc CPC III clusters in control and BMS-345541–treated samples (*n* = 6 per treatment, concatenated). Dashed circle identifies SnC CPC III cluster (cluster 16) that is depleted upon BMS-345541 treatment. (**B**) Heatmap of median expression of markers that are significantly different in the SnC CPC III cluster between BMS-345541–treated and DMSO-treated samples (paired *t* test, *P* < 0.05). (**C**) UMAP depicting the differential abundance of p16^ink4a^-expressing clusters in the combined control and BMS-345541–treated samples (*n* = 6). Dashed line encircles clusters significantly depleted upon BMS-345541 treatment (paired *t* test, *P* < 0.05). (**D**) UMAP of the median expression of p16^ink4a^ in the combined control and BMS-345541–treated samples (*n* = 6).

A shift in the density of chondrocyte clusters was noticeable in BMS-345541–treated samples (Figure 3, D–F). Clusters of the SOX9^hi^ group (clusters 11, 21, and 24) and select clusters of the SOX9^mid^ group (clusters 6, 7, 12, and16) were significantly depleted in cell numbers (Figure 3, D and E). In contrast, the clusters in the SOX9^lo^ group and select clusters of the SOX9^lo/mid^ group (clusters 3, 8, and 13) were significantly expanded (Figure 3, F and G). Another major effect of NF-κB pathway inhibition was a significant depletion of the senescent NOTCH1^+^STRO1^+^ CPC III population across all patient samples (Figure 4, A and B). No significant changes were observed in the abundance of Inf-A or Inf-D populations. Among the p16^ink4a^ clusters, however, there was a significant depletion of all clusters following BMS-345541 treatment except for SnC III (Figure 4, C and D). Thus, NF-κB pathway inhibition reduced inflammation and affected the abundance of multiple cell populations, including most p16^ink4a^ clusters.

### Kartogenin has a modest effect distinct from NF-κB pathway inhibition.

**Figure 5:**
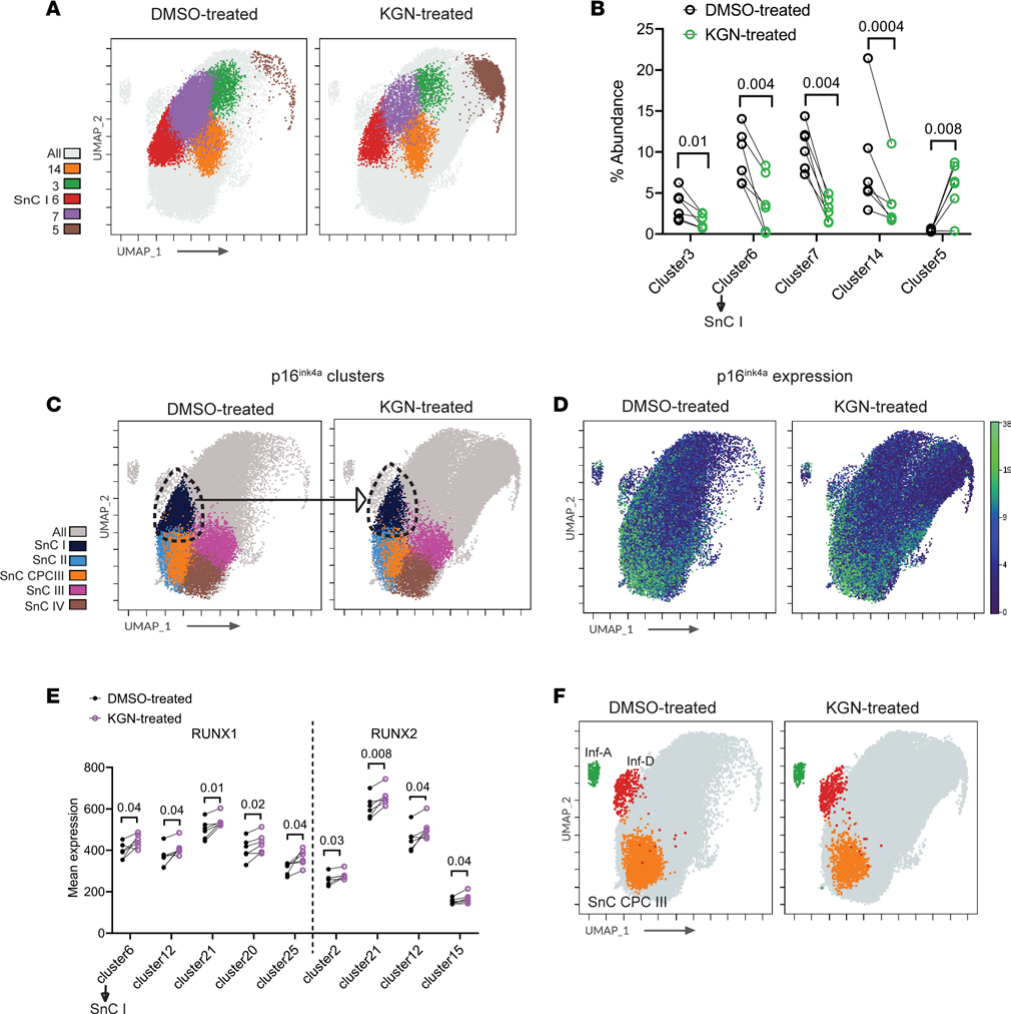
Effect of kartogenin treatment on the OA chondrocyte landscape. (**A**) UMAP of differentially abundant clusters in the combined control and kartogenin-treated samples (*n* = 6 per treatment, concatenated). (**B**) Percentage abundance of significantly different clusters in paired samples with control or kartogenin treatment. Statistical significance was calculated by paired *t* test at 95% confidence level and represented by adjusted *P* values. (**C**) UMAP of p16^ink4a^-expressing clusters in control and kartogenin samples. Dashed line encircling SnC I cluster represents significant depletion following kartogenin treatment. (**D**) UMAP of the median expression of p16^ink4a^ in the combined control and kartogenin-treated samples (*n* = 6 samples concatenated). (**E**) Mean expression of RUNX1 and RUNX2 in paired samples (*n* = 6) with control or kartogenin treatment. Statistical significance was calculated by paired *t* test at 95% confidence level and represented by adjusted *P* values. (**F**) UMAP illustrating Inf-A, Inf-D, and Snc CPC III clusters in control and kartogenin-treated groups (*n* = 6 samples concatenated).

Next, we examined the effect of a regenerative preclinical drug, kartogenin (18), on the OA landscape. In contrast to NF-κB pathway inhibition by BMS-345541, kartogenin treatment had a modest effect on the OA subpopulations (Figure 5A). Only 5 clusters showed a significant change in abundance (Figure 5B). One of the p16^ink4a^-expressing clusters in the SOX9^lo/mid^ group, SnC I (cluster 6), was significantly depleted (Figure 5, C and D). Other significantly depleted clusters included cluster 3 of the SOX9^lo/mid^ group and clusters 7 and 14 of the SOX9^mid^ group. Cluster 5 of the SOX9^lo^ group, consisting of low-inflammation chondrocytes, was the only cluster that showed significant expansion. RUNX2 expression was significantly downregulated in SnC I (cluster 6) among others (clusters 12, 20, 21, and 25) (Figure 5E). Mechanistically, kartogenin was shown to regulate the nuclear shuttling of core-binding factor β (CBFβ), which binds to RUNX family members (RUNX1, 2, and 3) to form a transcriptional complex that regulates multiple genes besides autoregulating the RUNX members themselves (18). Consistent with this mechanism, kartogenin treatment led to a significant increase in RUNX1 in clusters 2 and 15, while both RUNX1 and RUNX2 were increased in clusters 12 and 21. Excluding the SnC I population, none of the clusters where RUNX1 and/or RUNX2 were downregulated demonstrated a change in population density. Kartogenin did not affect the density of Inf-A, Inf-D, or SnC CPC III populations (Figure 5F). In the clusters grouped based on the median expression of SOX9 (Figure 1, C–F), NF-κB pathway inhibition by BMS-345541 significantly depleted the frequencies of the SOX9^mid^ and SOX9^hi^ groups, whereas treatment of OA chondrocytes with kartogenin had no significant effect in comparison with respective DMSO-treated controls (Supplemental Figure 4, A and B). Notably, both BMS-345541 and kartogenin increased the frequency of the SOX9^lo^ group (Supplemental Figure 4B). The median expression of SOX9 in individual clusters belonging to all groups was maintained in kartogenin-treated samples to levels comparable to those in DMSO-treated controls (Supplemental Figure 4C). In contrast, the SOX9 expression profile in BMS-345541–treated samples was dissimilar to that in DMSO- and kartogenin-treated counterparts, with lower median SOX9 expression levels in clusters belonging to the SOX9^mid^ and SOX9^hi^ groups (Supplemental Figure 4C), owing to the significant depletion in their frequencies.

### NF-κB inhibition modulates the secretome of OA chondrocytes to a greater degree than kartogenin.

As demonstrated by cyTOF, NF-κB pathway inhibition and kartogenin treatment had distinct effects on the OA landscape. To examine whether the drug treatments had a similar functional impact on the soluble mediators in the OA microenvironment, we sought to compare the effect of NF-κB pathway inhibition by BMS-345541 and kartogenin on the inflammatory secretome of OA chondrocytes. An 80-plex autoantibody assay (Luminex) comprising cytokines, chemokines, and growth factors was chosen to assess the effect of the drug treatments on the OA chondrocyte secretome.

**Figure 6:**
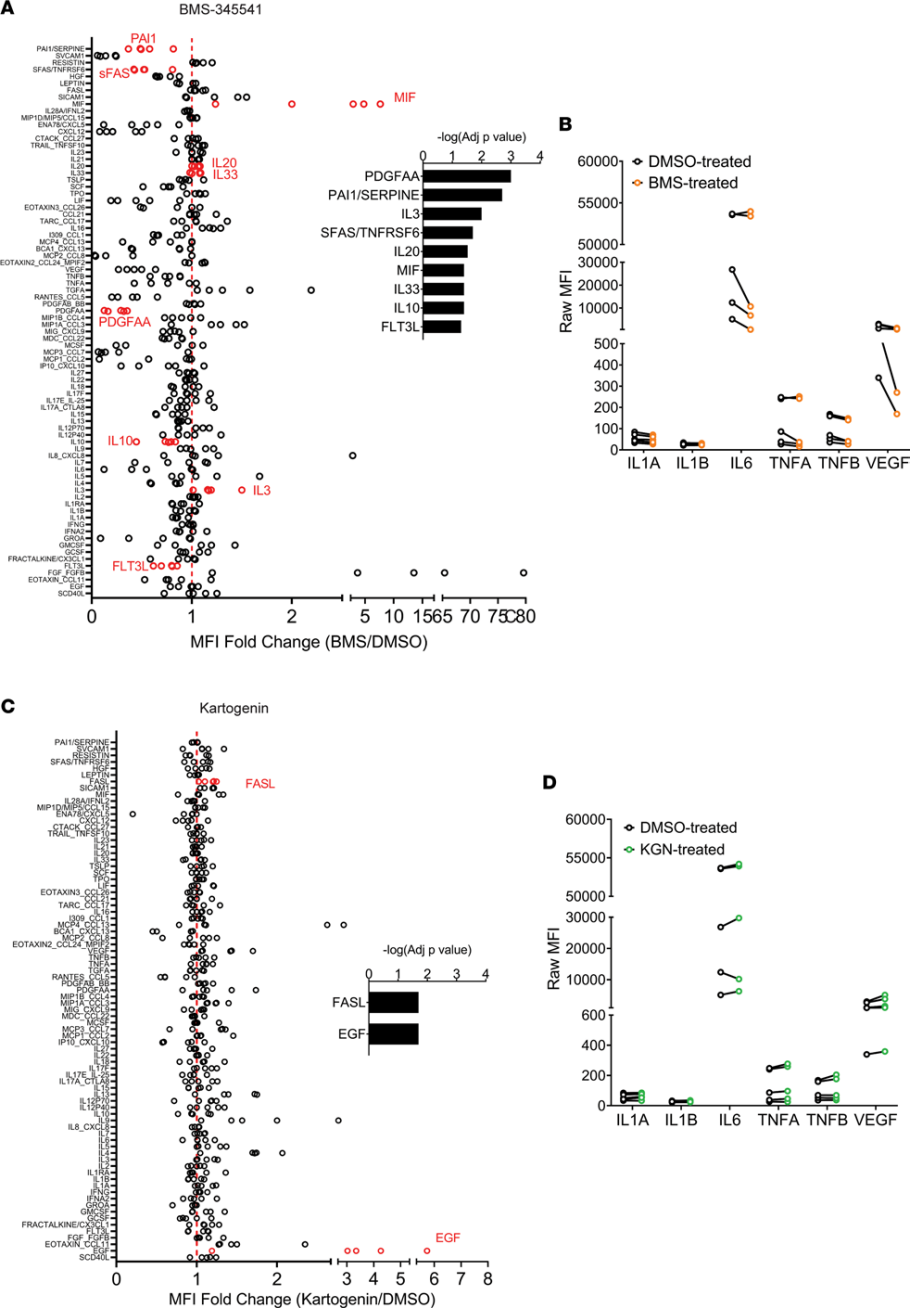
Effect of drugs on the OA secretory output. OA chondrocytes harvested from the surgical waste of 5 additional patients undergoing total knee replacement surgeries (*n* = 5) were treated with BMS-345541 (25 μM), kartogenin (25 μM), or DMSO for 48 hours. Spent medium was used for 80-plex autoantibody assay by Luminex. (**A** and **C**) Fold change in the raw mean fluorescence intensity (MFI) of all analytes in samples treated with BMS-345541 (**A**) or kartogenin (**C**) normalized to respective DMSO-treated samples. Statistical significance was measured by pairwise *t* test with post hoc Benjamini-Hochberg test. Significantly different (*P* < 0.05) analytes between drug treatment and DMSO controls are denoted in red, and log adjusted *P* values are provided (inset). (**B** and **D**) Raw MFI values of select SASP-associated analytes (IL-1α, IL-1β, IL-6, TNF-α, TNF-β, and VEGF) are represented in paired samples (*n* = 5) for BMS-345541 (**B**) and kartogenin (**D**) treatments.

To this end, OA chondrocytes were harvested from the surgical waste of 5 additional patients undergoing total knee replacement surgeries (*n* = 5 biological samples) and were treated with BMS-345541, kartogenin, or DMSO (vehicle control) for 48 hours, similar to the experimental conditions used for cyTOF study. Multiplex autoantibody assay by Luminex on the culture supernatants of the treatment groups revealed substantial dissimilarity in the secretory profile of BMS-345541–treated OA chondrocytes compared with kartogenin treatment and DMSO-treated controls (Figure 6 and Supplemental Figure 5). Notably, the levels of a plurality of secreted factors were decreased upon NF-κB pathway inhibition by BMS-345541 (Figure 6A). Significant fold decreases were observed in the levels of FMS-related tyrosine kinase 3 ligand (FLT3L), platelet-derived growth factor-AA (PDGF-AA), soluble FAS (sFAS), plasminogen activator inhibitor-1 (PAI1), and IL-10 when normalized to respective DMSO-treated controls. FLT3L, PDGF-AA, and sFAS are known to be accumulated in the synovial fluid in OA patients (19–21). PAI1 has pleiotropic roles dependent on tissue context such that it inhibits MMP activity under normal conditions whereas it increases ECM protein turnover, leading to fibrosis, under pathological conditions (22). IL-10 is an antiinflammatory cytokine that regulates TNF-α–mediated effects in OA cartilage (23). However, local fluctuations in IL-10 are transient but TNF-α expression levels can persist in OA (24); hence the downward trend in the levels of TNF-α and TNF-β in most patient samples implies positive anabolic regulation by BMS-345541 treatment (Figure 6B). Among the significantly elevated cytokines in BMS-345541–treated samples, the levels of macrophage migration inhibitory factor (MIF) were substantially higher compared with the levels observed in IL-20, -33, and -3. Contradictory roles have been defined for MIF in OA (25, 26), including induction of proinflammatory cytokines such as IL-1β as well as enhanced cell proliferation and antiapoptotic pathways (27, 28). The levels of cytokines implicated in senescence-associated secretory phenotype (SASP) such as IL-1β, IL-1α, IL-6, TNF-α, TNF-β, and VEGF did not change significantly upon BMS-345541 treatment, although the levels of IL-6 and VEGF were lowered in a majority of patient samples (Figure 6B). This response is likely due to the fact that BMS-345541 is not a senolytic and affected only a few senescent subpopulations. The secretory profile of kartogenin-treated samples did not deviate much from that of the respective DMSO-treated controls (Figure 6C). Notable exceptions were the significantly elevated levels of epidermal growth factor (EGF) and a modest increase in the death signaling receptor FasL. As with BMS-34551, the levels of select SASP-associated cytokines were variable between patient samples in kartogenin-treated samples (Figure 6D).

### Delineating responders and nonresponders.

**Figure 7:**
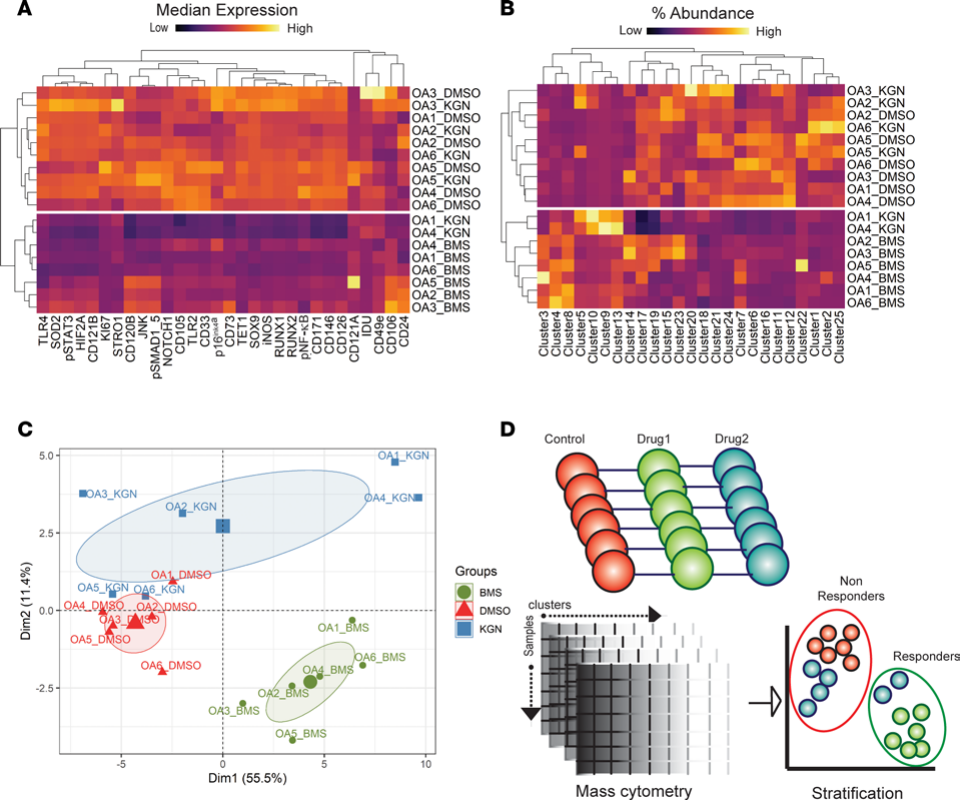
Delineating “responders” and “nonresponders” to OA drugs using cyTOF analyses. (**A**) Heatmap with hierarchical clustering of OA chondrocytes treated with control, BMS-345541, or kartogenin based on the expression profile of panel markers in each sample. (**B**) Heatmap with hierarchical clustering of OA chondrocytes treated with control, BMS-345541, or kartogenin based on the percentage abundance of clusters in each sample. (**C**) Principal component analysis plot of the distribution of patients based on cluster abundance and marker expression profiles in each patient sample with or without drug treatment. (**D**) Schematic outlining a platform for cyTOF-based profiling and analyses to identify responders and nonresponders to a drug candidate in patient cohorts.

A high-resolution platform like cyTOF can help provide precise insight into the patient-drug response. Using the response of the patients to the 2 drugs BMS-345541 and kartogenin, we sought to assess whether these drugs had a uniform effect on the patient-derived OA chondrocytes. Analyzing the effects of BMS-345541 treatment on the 25 distinct subpopulations in the patient chondrocytes, it was clear that this drug had a uniform effect. Unsupervised hierarchical clustering showed that the DMSO- and BMS-345541–treated OA chondrocytes fell into 2 distinct groups when accounting for expression of all markers (Figure 7A) and for cluster abundances (Figure 7B). On the other hand, kartogenin treatment led to a heterogeneous response among the patients even in this small cohort. Only 2 patients were responders, while, for the other patients, the control and kartogenin-treated samples clustered together (Figure 7, A and B). The principal component analysis plot mirrored the same findings (Figure 7C).

## Discussion

A high-resolution cellular atlas of OA articular cartilage is useful for understanding disease pathology and for devising new therapeutic strategies. We had previously identified multiple populations of CPCs and 2 rare chondrocyte subpopulations, Inf-A and Inf-D, that amplified or dampened inflammation (9). In this study, we have also utilized p16^ink4a^, which was previously identified to mark a functional senescent population in OA cartilage. Ablation or inhibition of p16^ink4a^-expressing cells had showed a decrease in SASP and delayed OA in a mouse model (11). We have identified 5 distinct clusters of p16^ink4a^-expressing senescent cells — SnC I, II, III, and IV along with the CPC III population that was earlier characterized by the high expression of NOTCH1 and STRO1. A recent study had also validated that progenitor cells tend to become senescent in OA cartilage (29). Besides CPC III, SnC III and IV were also identified to be senescent CPCs based on the high expression of both CD105 and p16^ink4a^. Notably, the Inf-A population was found to be distinct from the major p16^ink4a^-expressing senescent populations identified, although a few Inf-A cells showed p16^ink4a^ expression. These observations highlight the complex nature of the inflammatory microenvironment of cartilage, wherein multiple, small cell populations appear to be at play. Additionally, p16^ink4a^ is just one of the senescent markers, and the respective contributions of other markers including p21, p53, and uPAR to cartilage senescence have not been comprehensively analyzed (30). Incidentally, 3 of the SnC clusters (besides SnC III and IV) showed a coexpression of TLR2 and CD33 (Siglec 3) with p16^ink4a^. TLR2 has been previously observed to regulate oncogene-induced senescence and p16^ink4a^ function (31). The role of TLRs in addition to other players like p21, p53, and uPAR in cartilage-specific senescent cells and the multiple contributions to the SASP will be interesting to explore in future studies.

Another refinement to our previous analyses was that we did not exclude the SOX9^lo^ and SOX9^lo/mid^ groups of cells, which comprised 2.6% and 10.7% of the total cells. The SOX9^lo^ group clusters showed low expression levels of the CPC and inflammatory markers in the panel. These cells could be less inflamed OA chondrocytes or dedifferentiated chondrocytes. Although collagen 1 is a characteristic marker for dedifferentiated chondrocytes and collagen 10 for hypertrophic chondrocytes, these ECM markers did not stain well in isolated chondrocytes. It was, therefore, difficult to confirm the exact identity of this small population of cells.

Assaying population-level gene or protein expression changes elicited by drug treatments provides only a limited understanding of the effects of a drug on the cellular subpopulations, their crosstalk, and the cellular landscape that emerges after the drug treatment. For this study, we chose 2 model drug candidates — BMS-345541 and kartogenin — that had already been shown to be effective in the modulation of OA pathogenesis in an animal model (17, 32). While BMS-345541 selectively inhibits IκB kinase in the NF-κB pathway (14), kartogenin was originally identified in a screen to expand mesenchymal stem cells (18) and has since been shown in multiple studies to be a pro-chondrogenic modulator of OA progression in an animal model (32–34). The mode of action of these 2 drugs is distinct: while BMS-345541 dampens inflammation, kartogenin is pro-regenerative. Using these 2 drugs, we were able to identify the precise populations affected in OA chondrocytes. Interestingly, the targeted pathways were modulated only in select populations; for example, only about half the clusters showed significant downregulation of NF-κB signaling upon BMS-345541 treatment or RUNX 1/2 upregulation upon kartogenin treatment. While NF-κB pathway inhibition changed the overall landscape of the OA chondrocytes, affecting multiple populations, the effect of kartogenin was modest.

Interestingly, BMS-345541 not only affected overall NF-κB signaling and inflammation as expected but also reduced the abundance of p16^ink4a^-expressing senescent populations, including the NOTCH1- and STRO1-expressing SnC CPC III population. Kartogenin also reduced the frequency of SnC I but did not affect SnC CPC III or the other p16^ink4a^-expressing SnC populations. Neither BMS-345541 nor kartogenin depleted the inflammation-amplifying Inf-A or expanded the inflammation-dampening Inf-D populations. However, BMS-345541 treatment significantly decreased p–NF-κB expression in most clusters, including Inf-A (cluster 19) and Inf-D clusters (cluster 23). It was noted, however, that BMS-345541 treatment did not affect the secretion of CCL2 or CCL5, cytokines that were significantly reduced upon inhibition of activated p-JNK signaling in Inf-A populations (9), suggesting a difference in the functional effects of the NF-κB and JNK pathways operating in the Inf-A chondrocytes. An unexpected observation was that kartogenin treatment did not lead to the expansion of any CPCs. Instead, both kartogenin and BMS-345541 led to an expansion of clusters in the SOX9^lo^ group that did not show any CPC markers. It is important to discuss the potential effect of these drugs on nonpathogenic or relatively healthy cells in an arthritic joint. However, chondrocyte clusters in normal healthy donors are very different from those in OA patients, and these drugs will be tested and clinically used only in early or late OA patients. We characterized this in detail in our previous studies (9), where it was found that while some chondrocyte clusters were common among healthy and OA cohorts, many clusters present in healthy donors were lost in OA patients. Additionally, the OA cohort was enriched for highly inflamed CPC and non-CPC populations that were absent in healthy donors. In our data, we have observed that while NF-κB pathway inhibition, and to a modest degree kartogenin, leads to the depletion of a few senescent CPC clusters, most other clusters in OA chondrocytes are not depleted by these drugs.

The dynamic changes in the OA landscape inflicted by NF-κB pathway inhibition by BMS-345541 as observed by cyTOF were also reflected in the secretory profile of OA chondrocytes by 80-plex Luminex assay (Figure 6 and Supplemental Figure 5). A plurality of cytokines and chemokines were dampened by BMS-345541, while kartogenin had little effect on the OA secretory profile. While BMS-345541 had a variable effect on the levels of known SASP cytokines including IL-1α, IL-6, TNF-α, TNF-β, and VEGF, there was less discernible effect of kartogenin. This is expected since neither BMS-345541 nor kartogenin is a senolytic and they only affected a few p16^ink4a^ subpopulations.

Our results show that cyTOF-based analyses provide a powerful platform to test whether a drug elicits a uniform or a heterogeneous response in OA patients. The subset of patient samples tested here was grouped together in our previous cyTOF analyses in the absence of drugs, i.e., they belonged to the most abundant patient group, group A, and hence were a homogeneous set of patients (9). In testing of the 2 model drugs, it was, however, clear even in this small cohort (*n* = 6) that BMS-345541 treatment caused a uniform effect on all OA patients tested while only 2 patients responded to kartogenin. It was therefore apparent that with the use of cyTOF-based studies, patients can be divided into responders versus nonresponders based on a particular candidate drug, providing a proof of concept for this platform (schematic in Figure 7D). As a first step, we envision this platform to be useful in testing a new candidate drug on a Biobank of human OA cartilage samples (100–1,000 patients) and identify a potentially “uniform” response in patient populations. Using the treatment regimen here as an example, ex vivo treatment regimens for new drug candidates can be tested for efficacy and homogeneity of response. If a drug candidate is capable of a uniform response in end-stage patients (undergoing total joint replacement), it is likely to be beneficial in early and mid-stage OA patients as well. Since this step is completely missing in preclinical development right now, we believe that using a cyTOF-based platform can identify candidate drugs that show a heterogeneous response such that they can be discarded early in the drug discovery process, thus conserving millions of dollars in resources used in clinical trials.

The next step is to identify whether these candidate drugs will be useful for a subset of patients, i.e., stratify patients who are likely to benefit from a particular drug. Toward this goal, one possibility is a knee cartilage biopsy from an OA patient to test whether that patient is a good candidate for the biologic drug that can lead to joint repair and avoid total knee replacement. This approach, however, has its complications, since the biopsy is an invasive procedure and the benefits should outweigh the potential damage it can cause to cartilage and other joint tissues in a patient. Other methods are being investigated to stratify patients, like the identification of biomarkers in more accessible tissues, including blood, synovial fluid, and urine. Although future studies are needed to compare the relative merits of these putative approaches to patient stratification, their need is clear and urgent for devising effective drug discovery strategies for OA therapeutics. At a minimum, establishing an OA chondrocyte biobank for rapid in vitro preclinical drug screening could potentially identify drugs that are likely to fail in clinical trials owing to the heterogeneity in patient response. The limitations of this study include a small sample size of patients and a predetermined set of markers. While enlarging the patient cohort will enhance the clinical relevance of these observations, an expansion of the marker panel to include other cellular and signaling pathways can also broaden the understanding of the overall biological effect of the drugs in OA.

## Methods

### Isolation, culture, and drug treatment of OA chondrocytes.

OA cartilage tissue was procured from the surgical waste of patients (*n* = 6, >56 years of age, and a mix of male and female) undergoing total knee replacement who had radiographic end-stage OA, under an approved Institutional Review Board protocol (IRB35067). Cartilage was shaved off and chondrocytes were isolated and cultured as previously described (35). Briefly, to harvest chondrocytes from cartilage, the cartilage tissue was first dissected into smaller pieces and incubated in a dissociation medium (25 μg/mL collagenase II and 25 μg/mL collagenase IV in DMEM–F12 medium with 10% FBS) overnight in a CO_2_ incubator. After overnight treatment with the collagenases, the undigested tissue and debris were separated from the cells using a 70 μm cell strainer. The cells were centrifuged, washed twice with fresh DMEM–F12 medium, and plated in 150 mm tissue culture dishes. OA cells (*n* = 6 patient samples) at passage 2 were seeded at high density in 100 mm plates and treated with control (DMSO) or drug the next day for 48 hours. Drug doses were determined based on prior literature and validation: 25 μM NF-κB inhibitor BMS-345541 (Sigma-Aldrich B9935) (14, 17, 36) and 25 μM kartogenin (Sigma-Aldrich SML0370) (14–16).

### Cell staining and cyTOF.

OA cells treated with DMSO, BMS-345541, or kartogenin were stained with 25 μM 5-iodo-2′-deoxyuridine for 15 minutes at 37°C, followed by 0.5 μM cisplatin for 5 minutes at room temperature, fixed, washed, and frozen. Before staining, cells were thawed on ice and barcoded using the Cell-ID 20-plex Pd Barcoding Kit (Fluidigm). Individually barcoded cells were pooled together and labeled with antibodies conjugated with metal isotopes (Supplemental Table 1) as previously described (37). Cells were measured using the CyTOF 2 (Fluidigm) and injected using the Super Sampler. To normalize the signal over time during data acquisition, stained cells were resuspended in 10% EU beads (Fluidigm) in water before runtime.

### FlowSOM analysis and visualization.

Unsupervised hierarchical clustering by FlowSOM was performed on all live cells (cisplatin negative) per sample in Cytobank (Beckman Coulter Life Sciences) using all surface markers (CD24, CD126, CD121A, CD121B, CD120B, CD106, CD73, CD171, CD105, CD33, CD49E, CD146) in addition to SOX9, TET1, and HIF2A. The selection of markers for clustering approximated the methodology in the previous study (9) where all surface markers along with SOX9 and HIF2A were used so that previously defined subpopulations, particularly Inf-A, Inf-D, and CPC clusters, are adequately distinguishable. For consistency, 25 clusters and 10 metaclusters with 10 iterations were used as predetermined input parameters as calibrated and utilized in the previous study to identify subpopulations of interest with sufficient resolution (9). Downsampling was avoided, and therefore all cells per sample were included in creating self-organizing maps (SOMs) for cluster generation. Abundances of clusters were represented as a percentage of total cells analyzed per sample. UMAP projection was performed using Cytobank’s online platform to visualize FlowSOM-generated clusters using 8,043 cells per sample. The pheatmap package (https://cran.r-project.org/package=pheatmap) was used to generate heatmaps in RStudio software. Principal component analysis was performed using the factoextra package (http://www.sthda.com/english/rpkgs/factoextra) in RStudio based on all clusters and median expression of all markers in individual patient samples.

### Multiplex autoantibody assay.

OA chondrocytes from 5 new patient donors (different from those used in cyTOF experiment) were collected and treated with DMSO, BMS-345541 (25 μM), or kartogenin (25 μM) for 48 hours. Cell culture supernatants from all samples (DMSO, *n* = 5; BMS-345541, *n* = 5; kartogenin, *n* = 5) were collected and centrifuged at 10,000*g* for 10 minutes at 4°C to remove cell debris. The supernatants were snap-frozen in liquid nitrogen and submitted to the Human Immune Monitoring Center at Stanford University for multiplex autoantibody assay via 80-plex Luminex assay custom combination from EMD Millipore panel. Duplicate measurements for each analyte were recorded. Raw MFI values were averaged for each analyte and used in data analysis. Data visualization in the form of a heatmap of the average MFI readings for all 80 analytes across samples was conducted via the pheatmap package in R software.

### Statistics.

GraphPad Prism software was used for paired 2-tailed *t* tests for DMSO- and drug-treated samples corrected for multiple hypotheses by Bonferroni correction and reported as adjusted *P* values. In heatmaps, clustering was performed row- or column-wise using the Euclidean distance method for distance matrices. For Luminex assay, a pairwise *t* test was performed between BMS-4345541 or kartogenin and respective DMSO-treated samples with post hoc Benjamini-Hochberg test to avoid type 1 errors and represented as –log adjusted *P* values for significantly different analytes. All *P* values less than 0.05 were considered significant.

### Study approval.

This study was approved by the Stanford University Institutional Review Board (protocol IRB35067) with prior written informed consent from each patient.

## Author contributions

FCG and NB conceptualized the study. FCG designed the study and performed experiments. NS performed data analysis, experimentation, visualization, and data interpretation. NB and NS drafted the manuscript. FCG, NS, and NB reviewed and edited the manuscript.

## Supplementary Material

Supplemental data

## Version 1 10/04/2022

In-Press Preview

## Version 2 10/24/2022

Electronic publication

## References

[1] Mobasheri A (2017). The role of metabolism in the pathogenesis of osteoarthritis. Nat Rev Rheumatol.

[2] Rice SJ (2020). Interplay between genetics and epigenetics in osteoarthritis. Nat Rev Rheumatol.

[3] Parker E (2021). Multi-tissue epigenetic and gene expression analysis combined with epigenome modulation identifies RWDD2B as a target of osteoarthritis susceptibility. Arthritis Rheumatol.

[4] Rice SJ (2019). Prioritization of PLEC and GRINA as osteoarthritis risk genes through the identification and characterization of novel methylation quantitative trait loci. Arthritis Rheumatol.

[5] Guilak F (2018). Osteoarthritis as a disease of the cartilage pericellular matrix. Matrix Biol.

[6] Goldring SR, Goldring MB (2016). Changes in the osteochondral unit during osteoarthritis: structure, function and cartilage-bone crosstalk. Nat Rev Rheumatol.

[7] Chou C-H (2020). Synovial cell cross-talk with cartilage plays a major role in the pathogenesis of osteoarthritis. Sci Rep.

[8] Cheung P (2019). Single-cell technologies — studying rheumatic diseases one cell at a time. Nat Rev Rheumatol.

[9] Grandi FC (2020). Single-cell mass cytometry reveals cross-talk between inflammation-dampening and inflammation-amplifying cells in osteoarthritic cartilage. Sci Adv.

[10] Blüml S (2010). Antiinflammatory effects of tumor necrosis factor on hematopoietic cells in a murine model of erosive arthritis. Arthritis Rheum.

[11] Jeon OH (2017). Local clearance of senescent cells attenuates the development of post-traumatic osteoarthritis and creates a pro-regenerative environment. Nat Med.

[12] Wang Y (2020). Role of TLR2 and TLR4 in regulation of articular chondrocyte homeostasis. Osteoarthritis Cartilage.

[13] Price JS (2002). The role of chondrocyte senescence in osteoarthritis. Aging Cell.

[14] Burke JR (2003). BMS-345541 is a highly selective inhibitor of IκB kinase that binds at an allosteric site of the enzyme and blocks NF-κB-dependent transcription in mice. J Biol Chem.

[15] Ghosh S, Karin M (2002). Missing pieces in the NF-kappaB puzzle. Cell.

[16] Gao C (2021). Two reactive behaviors of chondrocytes in an IL-1β-induced inflammatory environment revealed by the single-cell RNA sequencing. Aging (Albany NY).

[17] McIntyre KW (2003). A highly selective inhibitor of I kappa B kinase, BMS-345541, blocks both joint inflammation and destruction in collagen-induced arthritis in mice. Arthritis Rheum.

[18] Johnson K (2012). A stem cell-based approach to cartilage repair. Science.

[19] Ramos MI (2013). FMS-related tyrosine kinase 3 ligand (Flt3L)/CD135 axis in rheumatoid arthritis. Arthritis Res Ther.

[20] Rosengren S (2010). Platelet-derived growth factor and transforming growth factor beta synergistically potentiate inflammatory mediator synthesis by fibroblast-like synoviocytes. Arthritis Res Ther.

[21] Hasunuma T (1997). Accumulation of soluble Fas in inflamed joints of patients with rheumatoid arthritis. Arthritis Rheum.

[22] Ghosh AK, Vaughan DE (2012). PAI-1 in tissue fibrosis. J Cell Physiol.

[23] Alaaeddine N (1999). Inhibition of tumor necrosis factor alpha-induced prostaglandin E2 production by the antiinflammatory cytokines interleukin-4, interleukin-10, and interleukin-13 in osteoarthritic synovial fibroblasts: distinct targeting in the signaling pathways. Arthritis Rheum.

[24] Struglics A (2015). Changes in cytokines and aggrecan ARGS neoepitope in synovial fluid and serum and in C-terminal crosslinking telopeptide of type II collagen and N-terminal crosslinking telopeptide of type I collagen in urine over five years after anterior cruciate ligament rupture: an exploratory analysis in the knee anterior cruciate ligament, nonsurgical versus surgical treatment trial. Arthritis Rheumatol.

[25] Rowe MA (2017). Reduced osteoarthritis severity in aged mice with deletion of macrophage migration inhibitory factor. Arthritis Rheumatol.

[26] Liu M (2021). Macrophage migration inhibitory factor may play a protective role in osteoarthritis. Arthritis Res Ther.

[27] Tillmann S (2013). Arrest functions of the MIF ligand/receptor axes in atherogenesis. Front Immunol.

[28] Song S (2022). Macrophage migration inhibitory factor family proteins are multitasking cytokines in tissue injury. Cell Mol Life Sci.

[29] Zhao X (2016). Leptin changes differentiation fate and induces senescence in chondrogenic progenitor cells. Cell Death Dis.

[30] Coryell PR (2021). Mechanisms and therapeutic implications of cellular senescence in osteoarthritis. Nat Rev Rheumatol.

[31] Hari P (2019). The innate immune sensor Toll-like receptor 2 controls the senescence-associated secretory phenotype. Sci Adv.

[32] Mohan G (2016). Kartogenin treatment prevented joint degeneration in a rodent model of osteoarthritis: a pilot study. J Orthop Res.

[33] Kwon JY (2018). Kartogenin inhibits pain behavior, chondrocyte inflammation, and attenuates osteoarthritis progression in mice through induction of IL-10. Sci Rep.

[34] Jing H (2019). Kartogenin preconditioning commits mesenchymal stem cells to a precartilaginous stage with enhanced chondrogenic potential by modulating JNK and β-catenin-related pathways. FASEB J.

[35] Taylor SEB (2014). A global increase in 5-hydroxymethylcytosine levels marks osteoarthritic chondrocytes. Arthritis Rheumatol.

[36] Murahashi Y (2018). Intra-articular administration of IκBα kinase inhibitor suppresses mouse knee osteoarthritis via downregulation of the NF-κB/HIF-2α axis. Sci Rep.

[37] Bendall SC (2011). Single-cell mass cytometry of differential immune and drug responses across a human hematopoietic continuum. Science.

