# Supplementary material for: A single-cell mass cytometry platform to map the effects of preclinical drugs on cartilage homeostasis
Source: JCI Insight. 2022 Oct 24;7(20):e160702. doi: 10.1172/jci.insight.160702 (PMC9744259; doi:10.1172/jci.insight.160702)
Supplement: Supplemental data [file jciinsight-7-160702-s008.pdf]

Supplemental Materials

Marker Types	Antibody	Isotope
Chondrocyte	SOX9	147
	CD24	160
Cell cycle	IDU	127
	Ki67	154
Inflammation	HIF2A	142
	pNF-κB(S29)	149
	iNOS	158
	CD126	163
	CD121B	143
	CD121A	156
	CD120B	165
Signaling	pJNK (T183/Y185)	175
	pSMAD1/5(S463/465)	176
	pSTAT3(pY705)	145
	TET1	148
	SOD2	144
	RUNX1	166
	RUNX2	167
CPC	CD106	159
	CD73	151
	NOTCH1	155
	STRO1	164
	CD171	170
	CD105	173
	CD33	168
	CD49E	169
	CD146	153
TLRs and Senescence	TLR4	162
	TLR2	171
	$p16^{ink4a}$	174

Supplemental Table 1. cyTOF marker panel. List of antibody markers categorized into chondrocyte, cell cycle, inflammation, signaling, chondroprogenitor (CPC) or TLRs, and senescence-related marker types with corresponding metal isotope conjugates identified by unique mass numbers.

Cluster Number	Group	Cluster Identity	Annotation
4,5,9,10	$SOX9^{low}$		
1,3,8,13,22	$SOX9^{low-medium}$		
2	$SOX9^{medium}$		
**6**		$CD33^{+}TLR2^{+}p16^{ink4a+}$	SnC I
7			
12			
14			
15			
**16**		$CD105^{+}NOTCH1^{+}STRO1^{+}$ $CD33^{+}TLR2^{+}p16^{ink4a+}$	SnC CPC III
**17**		$CD105^{+}p16^{ink4a+}$	SnC III
18			
**19**		$CD121A^{+}CD120B^{+}$	Inf-A
**23**		$3CD24^{+}$	Inf-D
25			
**11**	$SOX9^{high}$	$NOTCH1^{+}CD33^{+}TLR2^{+}p16^{ink4a+}$	SnC II
20			
21			
**24**		$CD105^{+}p16^{ink4a+}$	SnC IV

Supplemental Table 2. Table of high confidence cluster identities. Categorization of clusters based on their median SOX9 expression, and empirical identities, and annotation of select clusters using the markers included in the cyTOF panel are shown.

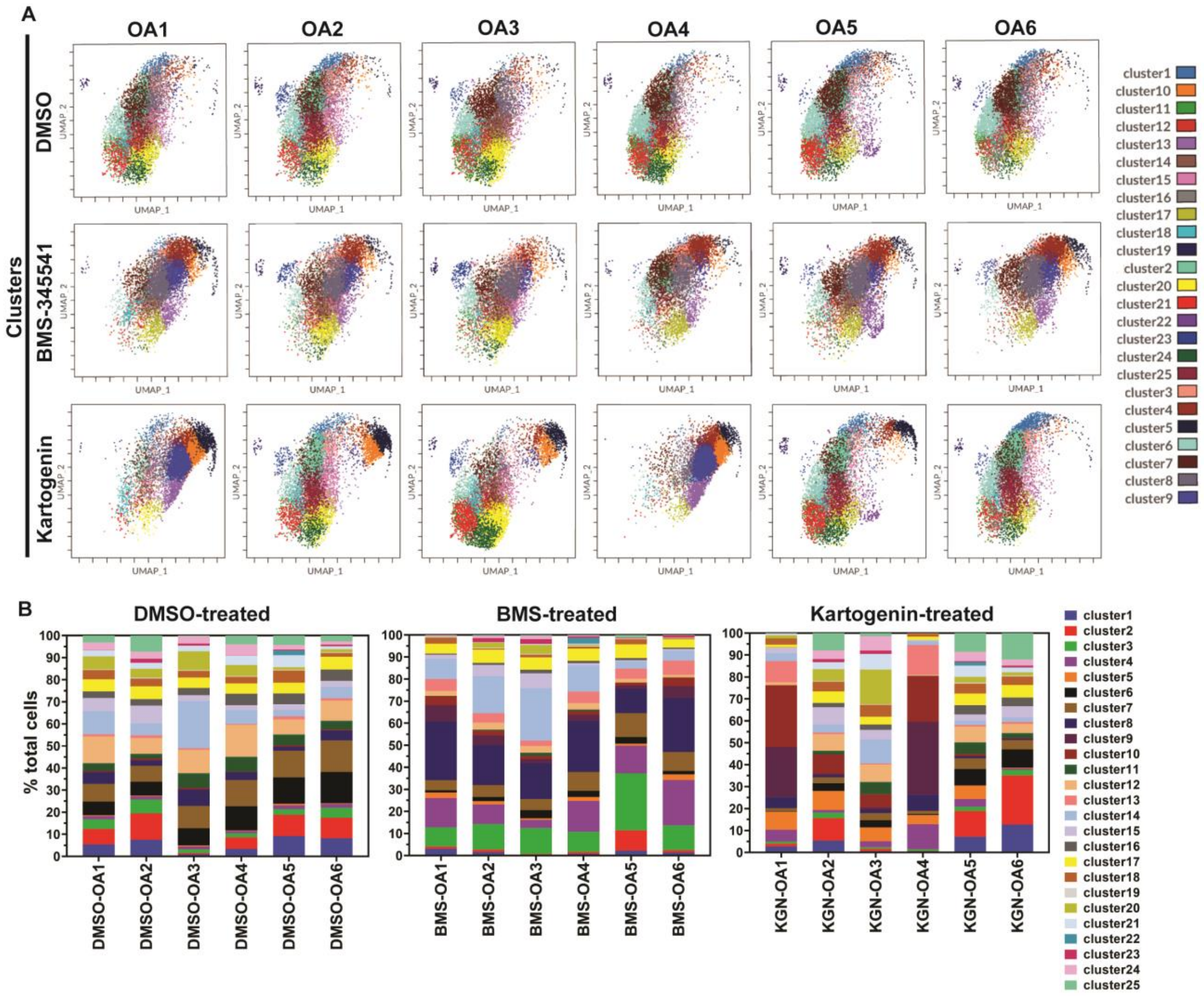

Supplemental figure 1. FlowSOM identified the OA chondrocyte landscape.
(A) UMAP of all 25 clusters identified by FlowSOM in individual DMSO, BMS-345541 and Kartogenin treated samples (n=6 per treatment). Each UMAP is a representation of 8043 cells. (B) Proportional representation of all clusters as a percent of total cells in each sample across treatment groups.

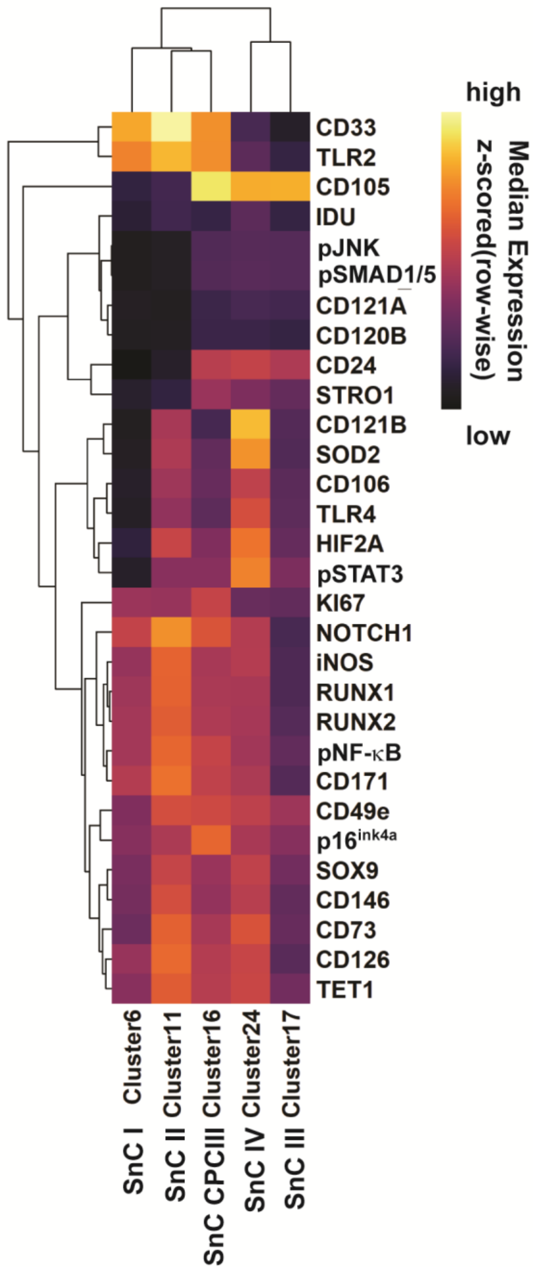

Supplemental figure 2. Median expression profile of $p16^{ink4a}$ clusters. Heatmap of the median expression of markers in the four $p16^{ink4a}$ clusters, namely SnC I, SnC II, SnC III, SnC CPC III, and Snc IV in combined DMSO-treated samples. The median intensities were z-scored row-wise (per marker) for standardization.

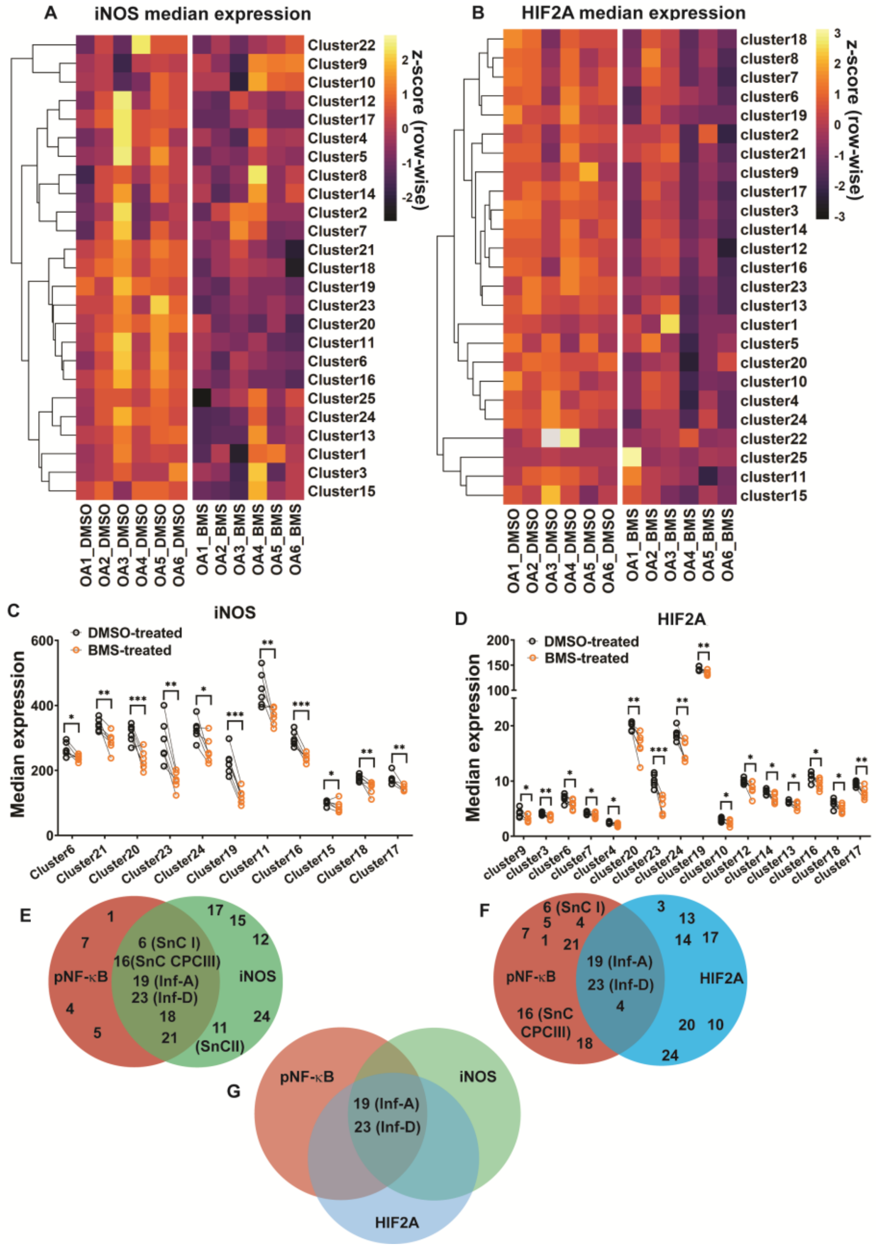

Supplemental figure 3. Effect of NF-κB pathway inhibition on iNOS and HIF2A expression.

Heatmap of the median expression of (A) iNOS and (B) HIF2A in all clusters between DMSO-treated controls and BMS-345541 -treated samples. The significant difference in the mean expression of iNOS or HIF2A was calculated by paired t-test at 95% confidence level. Venn diagram representing clusters where the expression of (C) pNF-κB and iNOS, (D) pNF-κB and HIF2A, and (E) pNF-κB, iNOS and HIF2A were commonly downregulated in BMS-345541 -treated samples when compared with DMSO-treated controls.

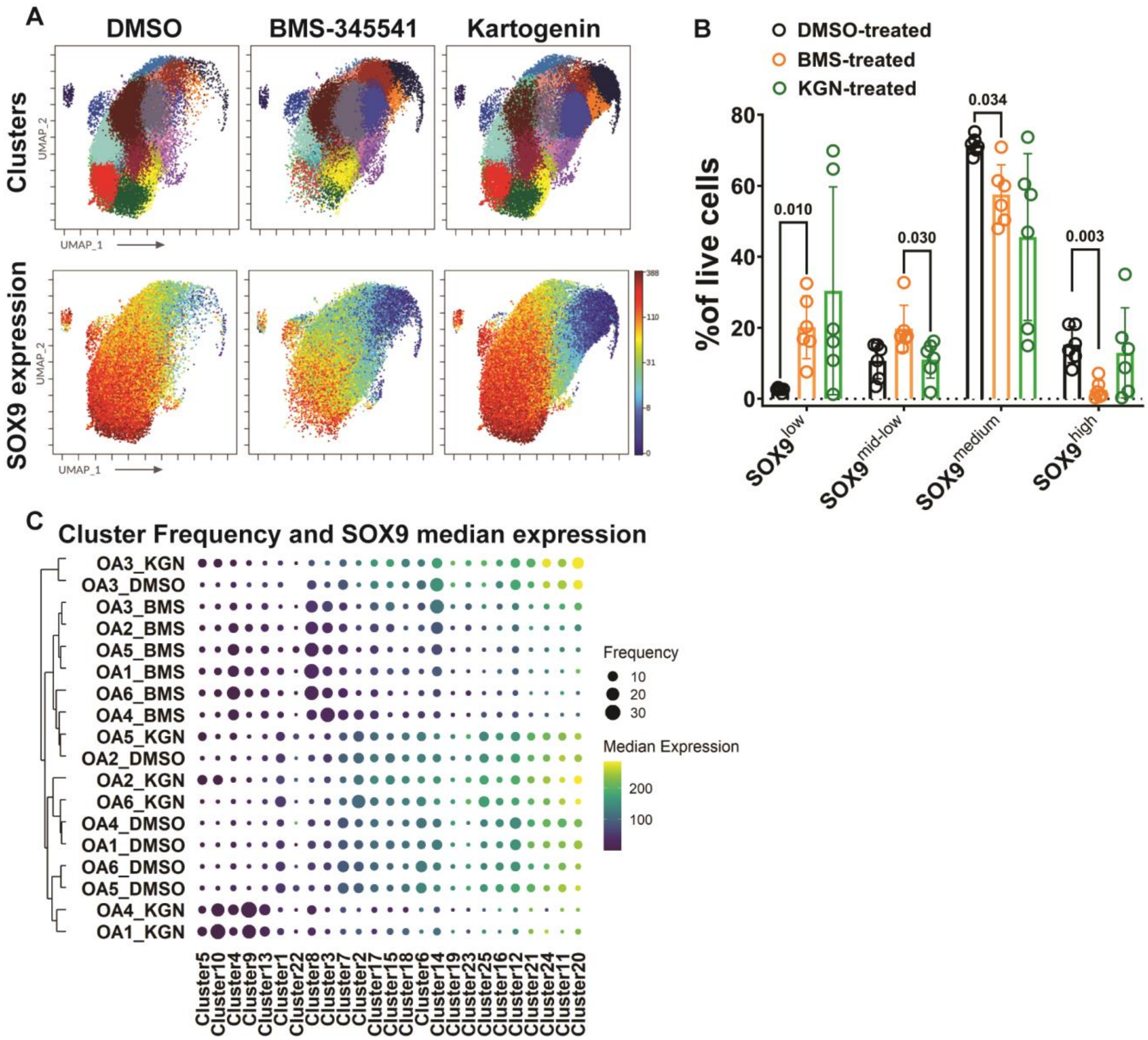

Supplemental figure 4. Drug effects on OA chondrocyte clusters stratified by SOX9 expression.

(A) UMAP of all clusters juxtaposed below with the median expression of SOX9 in the clusters in concatenated (n=6) DMSO, BMS-345541 and Kartogenin treated samples. (B) Frequency of $SOX9^{low}$, $SOX9^{low\text{-}medium}$, $SOX9^{medium}$ and $SOX9^{high}$ clusters in drug-treated groups. Bar graphs represent mean ± standard deviation. Statistical significance was calculated by repeated measures one-way ANOVA with Bonferroni correction and significant differences were represented by adjusted p values. (C) Clustered dot plot represents the frequency as a percent of total cells in each cluster (dot size) and the median SOX9 expression in each cluster (dot color) in individual samples.

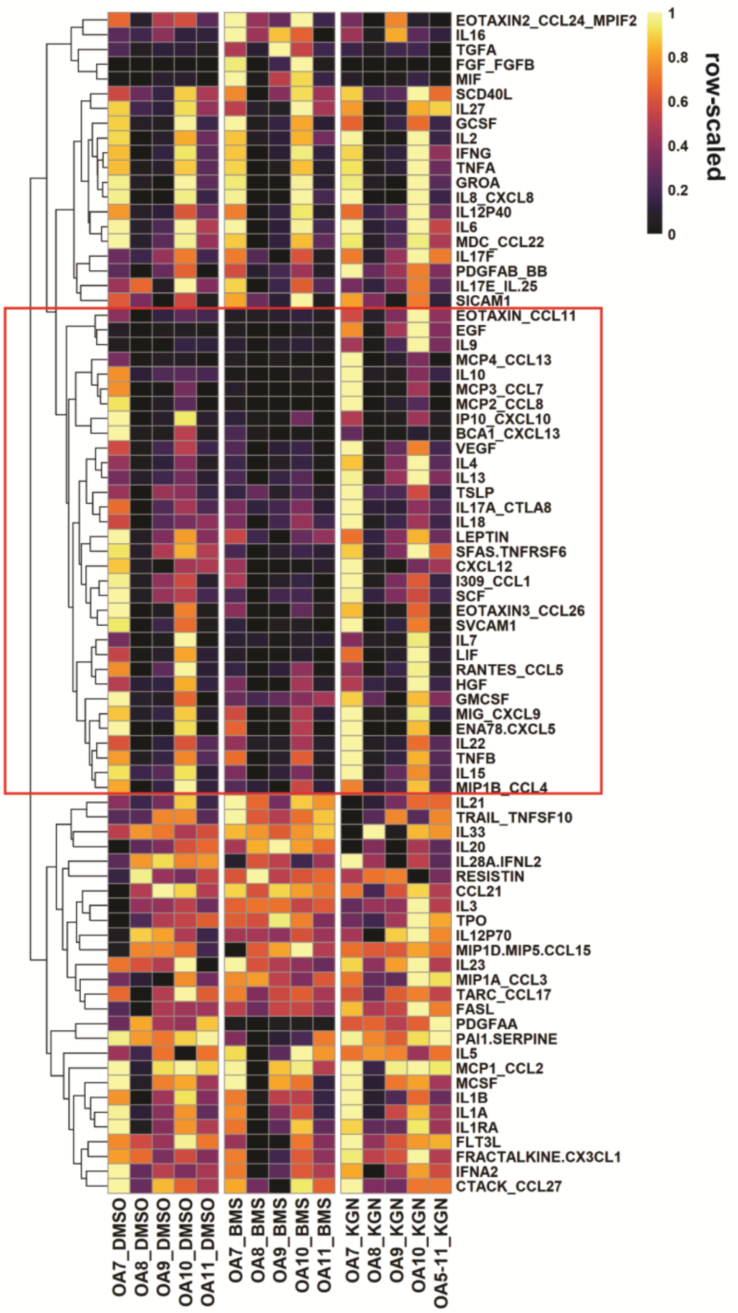

Supplemental figure 5. Drug treatment effects on the secretory profile of OA chondrocytes. Heatmap represents the raw MFI of 80 analytes (scaled row-wise) measured by multiplex autoantibody assay by Luminex in all DMSO, BMS-345541 and Kartogenin treated samples. Hierarchical clustering of rows as a measure of Euclidean distance was performed to visualize treatment effects on analytes. Analytes that are dampened in BMS-345541 treatment group compared to DMSO or Kartogenin treatment groups cluster together and are indicated by rectangle box in red.
